# Complete genome sequence of *Varibaculum* sp. GTC20041 isolated from a clinical specimen in Japan

**DOI:** 10.1128/mra.00670-25

**Published:** 2025-09-12

**Authors:** Masahiro Hayashi, Jun Yonetamari, Yoshinori Muto, Kaori Tanaka

**Affiliations:** 1 Institute for Glyco-core Research iGCORE, Gifu University 12785, Gifu City, Gifu, Japan; 2 Division of Anaerobe Research, Life Science Research Center, Gifu University 12785, Gifu City, Gifu, Japan; 3 Gifu University Center for Conservation of Microbial Genetic Resource, Gifu, Japan; 4 Division of Clinical Laboratory, Gifu University Hospital 476117, Gifu, Japan; 5 United Graduate School of Drug Discovery and Medical Information Sciences, Gifu University, Gifu City, Gifu, Japan; 6 Center for One Medicine Innovative Translational Research (COMIT), Gifu University 12785, Gifu, Japan; Loyola University Chicago, Chicago, Illinois, USA

**Keywords:** genomes, *Varibaculum*, anaerobes

## Abstract

We report the complete genome sequence of *Varibaculum* strain GTC20041 isolated from a clinical specimen in Japan. The genome comprised a circular chromosome with a length of 2,266,073 bp.

## ANNOUNCEMENT

*Varibaculum* is a gram-positive, facultatively anaerobic, nonspore-forming, and nonmotile bacterial genus from the family *Actinomycetaceae. Varibaculum* species are commonly found in various human samples (1, 2). In particular, strain GTC20041 was identified in a human clinical specimen in Aichi, Japan, in 2021. More specifically, it was isolated from the ascites of an adult patient via anaerobic culture from an ascites sample submitted for clinical testing. The strain was maintained on Brucella HK agar supplemented with 5% laked sheep blood at 37°C for 48 h under anaerobic conditions (82% N_2_, 10% CO_2_, and 8% H_2_). The strain was repeatedly subcultured three times under the same conditions for purification. The present study was conducted in accordance with the provisions of the Declaration of Helsinki. Identification via matrix-assisted laser desorption/ionization time-of-flight mass spectrometry (MALDI-TOF MS) (MALDI Biotyper, Bruker, Germany 12438 MSP) in our laboratory confirmed the classification of strain GTC20041 solely as *Varibaculum* at the genus level.

 To obtain the complete genome sequence, the genome of GTC20041 was sequenced using PromethION 2i (Oxford Nanopore Technologies, Oxford, UK) for long-read sequencing and the NovaSeq 6000 system (Illumina Inc., USA) for short-read sequencing as previously described (3–5). Genomic DNA was extracted from the pellet using NucleoBond HMW DNA (MACHEREY-NAGEL, Japan) according to the manufacturer’s instruction. For long-read sequencing, a library was constructed using a Native Barcode Sequencing Kit (SQK-NBD114-24; Oxford Nanopore Technologies) without shearing, followed by the removal of small fragments using a Short Read Eliminator XS (Circulomics, Baltimore, MD, USA). Sequencing was performed using a FLO-PRO114M flow cell. The MinkNOW v.23.11.8 software was used for base calling and raw reads were trimmed and quality filtered using NanoFilt v.2.7.1 (6) with the parameters "-l 1000 -q 10 -headcrop 50." For short-read sequencing, the DNA Prep (M) Tagmentation Kit (Illumina Inc.) was used for library construction. Subsequently, 2 × 151 bp paired-end sequencing was performed using the NovaSeq 6000 platform. Raw sequencing reads were processed using fastp v.0.20.1 (7) with the parameters "-q 30 n 20 t 1 T 1." The quality of short reads was also assessed using fastp v.0.20.1 (7). The mean read quality of long reads was scored using NanoPlot 1.32.1 (7). Short (>91.8% of bases > Q30 averaged) and long reads (mean read quality of 15.9) were assembled using Unicycler v.0.4.8 (8) with default settings. In particular, assembly circularization and rotation were performed automatically. The assembly was rotated to start with the *dnaA* gene on the forward strand. Annotation of the assembled genomes was performed using the DDBJ Fast Annotation and Submission Tool (DFAST) (https://dfast.ddbj.nig.ac.jp/) (9). Average nucleotide identity (ANI) analysis was conducted using PyANI v.0.2.12 (10).

 Table 1 summarizes the genomic information. Quality assessment and genome statistics were computed using CheckM (v.1.2.2) (11) and seqKit (v.2.9.0) (12), respectively. CheckM indicated that the genome was 99.05% complete with 0.71% contamination. DFAST predicted 1,937 coding sequences, six ribosomal RNAs, 49 transfer RNAs, and 2 CRISPR sequences.

**TABLE 1:** Information on the complete genome sequence of a *Varibaculum* sp. strain isolated from a human clinical specimen in Japan

	Strain name
Parameter	*Varibaculum* sp. GTC20041
NovaSeq6000 sequencing^*a*^	
No. of reads	6,577,930
Size (kb)	983,073
Avg coverage (×)	434
DRA accession no.	DRR683174
ONT sequencing^*a*^	
No. of reads	213,594
Size (kb)	800,004
Avg read length (bp)	7,249
Avg coverage (×)	353
N50	7,318
DRA accession no.	DRR683173
Assembly	
Assembly N50 (bp)^*c*^	2,266,073
Estimated genome completeness (%)^*d*^	99.05
Estimated genome contamination (%)^*d*^	0.71
Genome structure	1 chromosome
DDBJ/GenBank accession no.	AP041144
Genome size (bp)	2,266,073
	
GC content (%)	
(chromosome/plasmid name)	52.6
	
No. of coding sequences^*b*^	1,937
Number of rRNAs^*b*^	6
Number of tRNAs^*b*^	49
Number of CRISPRs^*b*^	2

^*a*^ DRA, DDBJ Sequence Read Archive.

^*b*^ DFAST, DDBJ Fast Annotation and Submission Tool.

^*c*^ Determined with seqkit v2.3.0.

^*d*^ Determined with CheckM v1.2.2.

 BLASTN analysis (13) of the 16S rRNA gene sequence identified *Varibaculum cambriense* CCUG 44998^T^ (Acc.no. AJ428402) as the closest match, with 98.6% identity. However, species identification via the genome sequence using GTDB-tK (v.2.4.0) was unsuccessful (14). ANI analysis of the complete genome sequence revealed that GTC20041 is most closely related to *V. cambriense* (ASM42006v1), with an ANI value of 88.1%. Based on these results, we could not assign the strain to a species at this time.

## Data Availability

The genome sequence of Varibaculum sp. (GTC20041) has been deposited into DDBJ (https://www.ddbj.nig.ac.jp/index.html) under the accession number AP041144. Raw sequence data for GTC20041 have been deposited into the DDBJ/Sequence Read Archive under the accession numbers DRR683173 and DRR683174.
